# Supplementary material for: Supporting children and young people when making decisions about joining clinical trials: qualitative study to inform multimedia website development
Source: BMJ Open. 2019 Jan 9;9(1):e023984. doi: 10.1136/bmjopen-2018-023984 (PMC6340013; doi:10.1136/bmjopen-2018-023984)
Supplement: Supplementary data [file bmjopen-2018-023984supp001.pdf]

Appendix 1: Participant characteristics

Children and Young people			
**Participant ID**	**Gender**	**Age (years)**	**Experience of being approached about a children's trial**
CYP/16	Male	10	Yes
CYP/18	Female	18	Yes
CYP/19	Female	16	No
CYP/20	Male	18	No
CYP/21	Female	15	No
CYP/23	Female	16	Yes
CYP/25	Male	9	Yes
CYP/29	Female	12	No
CYP/31	Female	19	Yes
CYP/35	Female	16	No
CYP/39	Female	14	No
CYP/43	Male	9	Yes
CYP/44	Male	6	No
CYP/47	Male	6	No
CYP/49	Male	7	Yes
CYP/51	Female	15	Yes
CYP/53	Female	16	No
CYP/55	Female	6	No
CYP/57	Female	6	Yes
CYP/59	Male	13	No
CYP/62	Female	8	Yes
Parents			
**Participant ID**	**Gender**	**Experience of being approached about a children's trial**	
Parent/17	Female	Yes	
Parent/22	Female	Yes	
Parent/24	Female	Yes	
Parent/26	Female	Yes	
Parent/27	Male	Yes	
Parent/28	Male	No	

Parent/30	Female	No
Parent/32	Female	No
Parent/33	Male	No
Parent/34	Female	No
Parent/36	Female	No
Parent/37	Female	Yes
Parent/38	Female	No
Parent/41	Female	Yes
Parent/42	Male	Yes
Parent/46	Female	Yes
Parent/48	Male	Yes
Parent/50	Female	Yes
Parent/52	Male	No
Parent/54	Female	No
Parent/56	Female	No
Parent/58	Male	Yes
Parent/60	Male	No
Parent/61	Female	Yes
Professionals		
**Participant ID**	**Gender**	**Current role**
Professional/1	Female	Paediatric rheumatology consultant
Professional/2	Female	Paediatric rheumatology consultant
Professional/3	Female	Paediatric renal consultant
Professional/4	Female	Paediatric intensive care nurse
Professional/5	Female	Operations manager
Professional/6	Female	Paediatric research nurse
Professional/7	Female	Research governance (previous researcher)
Professional/8	Female	Research pharmacist
Professional/9	Female	Research nurse
Professional/10	Female	Research nurse
Professional/11	Male	Research nurse
Professional/12	Female	Research nurse
Professional/13	Female	Data manager (involved in recruitment)

Professional/14	Male	Paediatric respiratory consultant
Professional/15	Male	Paediatric respiratory consultant
Professional/40	Female	Play specialist
Professional/45	Male	Paediatric pharmacist
